# Caffeoyl–Pro–His amide relieve DNCB-Induced Atopic Dermatitis-Like phenotypes in BALB/c mice

**DOI:** 10.1038/s41598-020-65502-2

**Published:** 2020-05-21

**Authors:** Sunhyae Jang, Jungyoon Ohn, Ji Won Kim, So Min Kang, Dasom Jeon, Chan Yeong Heo, Yoon-Sik Lee, Ohsang Kwon, Kyu Han Kim

**Affiliations:** 1 Laboratory of Cutaneous Aging and Hair Research, Clinical Research Institute, Seoul National University Hospital, Seoul, Republic of Korea; 2 Institute of Human Environment Interface Biology, Seoul National University, Seoul, Republic of Korea; 3 Department of Dermatology, College of Medicine, Seoul National University, Seoul, Republic of Korea; 4 Department of Plastic and Reconstructive Surgery, College of Medicine, Seoul National University, Seoul, Republic of Korea; 5 Department of Plastic and Reconstructive Surgery, Seoul National University Bundang Hospital, Gyeonggi-do, Republic of Korea; 6 School of Chemical and Biological Engineering, Seoul National University, Seoul, Republic of Korea

**Keywords:** Drug discovery and development, Atopic dermatitis

## Abstract

The main factors involved in the pathogenesis of atopic dermatitis (AD) are skin barrier abnormality, allergy/immunology, and pruritus. Considering how oxidative stress influences these factors, antioxidant agents may be effective candidates in the treatment of AD. To evaluate the effect of Caffeoyl–Pro–His amide (CA-PH), an antioxidant agent, on 2,4-dinitrochlorobenzene (DNCB)-induced AD-like phenotypes in BALB/c mice. Topical sensitization and challenge by DNCB were performed on the dorsal skin of BALB/c mice to induce AD-like cutaneous lesions, phenotypes, and immunologic response. CA-PH was applied topically for 2 weeks to assess its effects on DNCB-induced AD-like phenotypes. As a result, CA-PH relieved DNCB-induced AD-like phenotypes quantified by dermatitis severity score, scratching duration, and trans-epidermal water loss. Histopathological analysis showed that CA-PH decreased epidermal thickening, the number of mast cells, and eosinophil infiltration in dermis. Immunohistochemical staining revealed that CA-PH recovered skin barrier-related proteins: filaggrin, involucrin, and loricrin. As for the immunologic aspects, CA-PH treatment lowered mRNA or protein levels of interleukin (IL)-4, IL-6, IL-17a, IL-1b, IL-31, and IL-33 levels and thymic stromal lymphopoietin (TSLP) levels in cutaneous tissue, reducing the DNCB-induced serum IgE level elevation. In conclusion, topical CA-PH may be a therapeutic option for the treatment of AD.

## Introduction

Atopic dermatitis (AD) is a pruritic cutaneous inflammatory disorder with chronic recurrence. AD affects up to 25% of children and 3% of adults^1^. AD can be controlled with topical and/or systemic treatments, but some cases are resistant to conventional therapies^2^. Its pathogenesis is characterized by reciprocal interactions between the three major factors in vicious cycle: pruritus, skin barrier abnormality, and immunologic dysregulation^3^. All three factors are influenced by oxidative stress, an imbalance between production and scavenging capacity of reactive oxygen species (ROS) in the tissue, resulting oxidative stress can develop or aggravate AD^4–11^. Oxidative stress is associated with itching sense, thus, suppressing oxidative stress attenuates acute and chronic pruritus^12^. In addition, ROS can induce skin barrier dysfunction^13^ and impact on cutaneous immunology by inducing helper type 2 T cell (Th2) responses with oxidized lipids and thymic stromal lymphopoietin (TSLP) in epithelial cells^14^. The increased TSLP expression has been attributed to the pathogenesis of AD^15^. In this context, reducing ROS in the cutaneous tissue may be an efficient strategy to regulate the three major factors of AD pathogenesis and alleviate AD-associated phenotypes.

**Figure 1:**
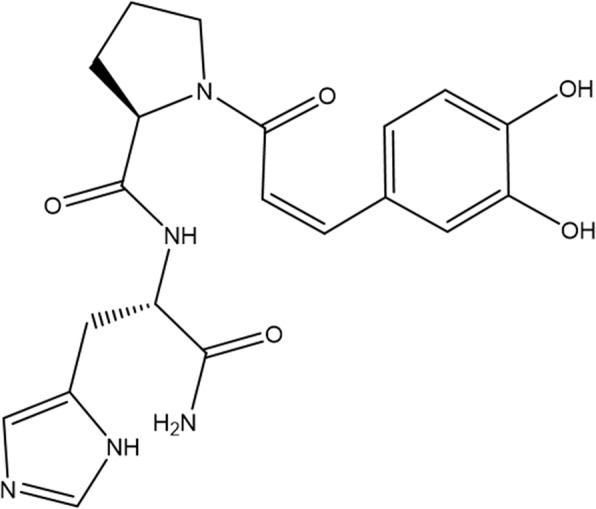
Caffeoyl-prolyl-histidine amide (CA-L-Pro-L-His-NH_2_; CA-PH).

Caffeic acid is a major subgroup of phenolic compounds with antioxidant effect^16,17^. Kwak *et al*. developed a conjugated peptide form of caffeic acid, caffeoyl-prolyl-histidine amide (Fig. 1 CA-L-Pro-L-His-NH_2_; CA-PH), to enhance anti-oxidative property with sufficient stability^18,19^. Proline in this conjugated form enhances the antioxidant effect, while the imidazole ring of histidine optimizes the effect. Subsequent study on the structural profits of CA-PH showed that it reduced ROS and increased the expression of heme oxygenase (HO-1)^20^. Considering that the enhancement of HO-1 reduces the development of AD skin lesions in mice^21^, and caffeic acid is not cytotoxic under experimental conditions up to high concentration (1 mM)^22^, CA-PH may be a therapeutic candidate to improve AD phenotypes. In this study, we evaluated the effect of CA-PH on the 2,4-dinitrochlorobenzene (DNCB)-induced AD-like phenotypes in BALB/c mice.

## Results

### CA-PH reduced the dermatitis score and scratching behavior duration

**Figure 2:**
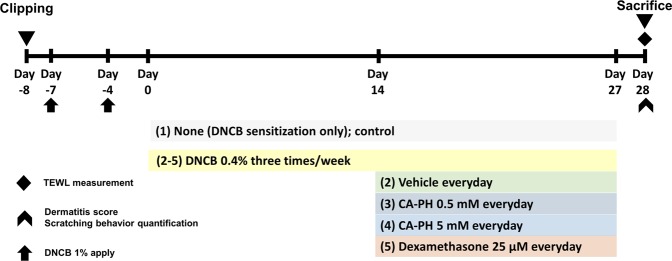
Experiment timeline. Cutaneous 2,4-dinitrochlorobenzene (DNCB) sensitization was performed by applying 1% DNCB on the dorsal skin of the mice at Day -7 and Day -4. The DNCB-sensitized BALB/c mice were divided into five groups: (1) DNCB sensitization only (2) DNCB-induced atopic dermatitis (AD) + vehicle, (3) DNCB-induced AD + Caffeoyl-Prolyl-Histidine amide (CA-PH) 0.5 mM, (4) DNCB-induced AD + CA-PH 5 mM, and (5) DNCB-induced AD + topical dexamethasone 25 µM. To induce AD-like phenotypes, DNCB was topically applied on the mice three times a week for 4 weeks (Day 0 to Day 27) in groups 2, 3, 4, and 5. In each group, vehicle, CA-PH 0.5 mM, or 5 mM, dexamethasone 25 µM was topically applied to the mouse dorsal skin daily for 2 weeks (Day 14–27). The dermatitis score, scratching behavior, trans-epidermal water loss (TEWL) were measured, and mice were sacrificed for tissue analysis at Day 28.

**Figure 3:**
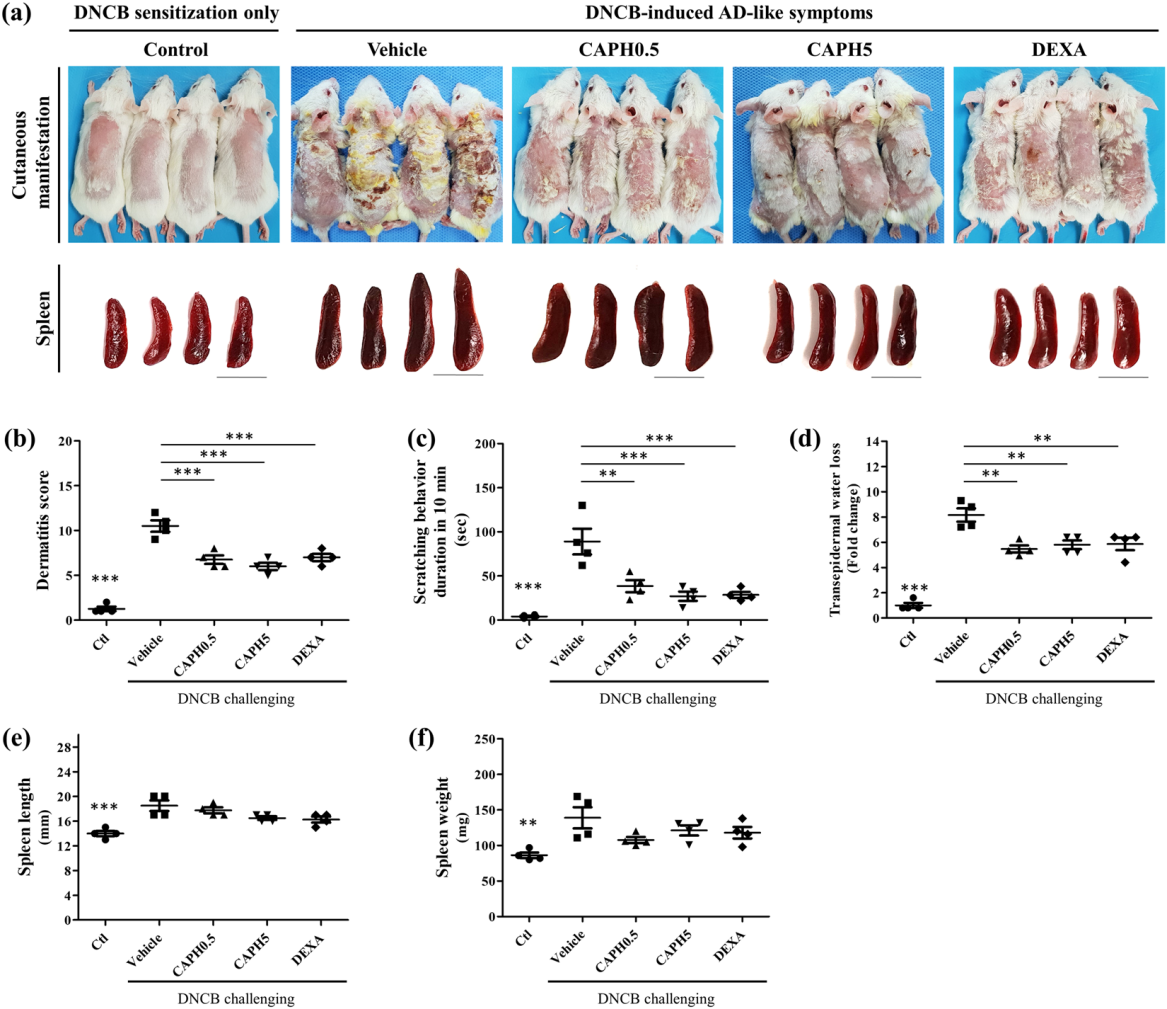
Effect of CA-PH on cutaneous manifestations and spleen of DNCB-induced AD-like phenotypes in BALB/c mice. (**a**) Cutaneous manifestations (upper panel) and spleen (lower panel, bar: 1 cm) of mice in each group at Day 28. (**b**) Dermatitis score was quantified based on the erythema, edema/papulation, excoriation, and scaling/dryness. Caffeoyl-Prolyl-Histidine amide (CA-PH) (0.5 mM and 5 mM) reduced DNCB-induced increased dermatitis score significantly. (**c,d**) Scratching behavior duration and trans-epidermal water loss significantly increased in vehicle-treated mice compared to the control group, which were decreased by CA-PH treatment. (**e,f**) The length and weight of spleen significantly increased in the vehicle group, which was slightly decreased by the CA-PH without statistical significance (data are presented as the mean ± SE; *p < 0.05, **p < 0.01, and ***p < 0.001).

To induce AD-like cutaneous condition in mouse, we used cutaneous DNCB sensitization and challenging in BALB/c mice (Fig. 2). On Day 28, the dorsal skins of mice applied with DNCB and vehicle showed prominent erythema, edema/papulation, excoriation, and scaling/dryness compared to the control group (dermatitis score: 10.50 ± 0.65 *vs*. 1.25 ± 0.25; *p* < 0.001) (Fig. 3a,b), indicating that DNCB efficiently induced AD-like phenotypes. CA-PH significantly attenuated the dermatitis severity in both the CA-PH-treated groups compared to the vehicle group (6.75 ± 0.48 in the 0.5 mM group or 6.00 ± 0.41 in the 5 mM group *vs*. 10.50 ± 0.65 in the vehicle group; *p* < 0.001) (Fig. 3a,b).

The duration of scratching behavior was quantified. The duration significantly increased in vehicle-treated mice compared to the control (4.0 ± 0.9 s *vs*. 89.0 ± 14.7 s; *p* < 0.001), which declined by applying CA-PH. The duration of scratching behavior was 38.5 ± 6.9 s and 27.0 ± 5.2 s in the CA-PH 0.5 mM and 5 mM groups, respectively (*p* < 0.01 and *p* < 0.001 compared to the vehicle group) (Fig. 3c). Thus, CA-PH reduced DNCB-induced AD-like phenotypes in BALB/c mice, in terms of eczematous lesion and pruritus.

There was a significant increase in both the length and weight of the spleen in vehicle-treated mice (Fig. 3e,f). Compared to the vehicle-treated group, the length and weight of spleen of the CA-PH-treated groups showed lower value but without statistical significance.

### CA-PH restored skin barrier function

TEWL is elevated in AD patients, reflecting skin barrier dysfunction^23,24^. In this regard, we checked TEWL of mice in each group. The value of each group was represented as fold change compared to control. TEWL of the vehicle group was 8.16 ± 0.53 folds of the control group (*p* < 0.001). The disrupted skin barrier function, as a DNCB-induced AD like symptom, was restored by CA-PH. The CA-PH-treated groups showed significantly lower TEWL than that of the vehicle-treated mice group, suggesting that CA-PH improved skin barrier function (5.47 ± 0.28 folds in 0.5 mM; and 5.8 ± 0.35 in 5 mM; *p* < 0.01 compared to vehicle group) (Fig. 3d).

### CA-PH reduced cutaneous epidermal lichenification and ROS level in cutaneous tissue

**Figure 4:**
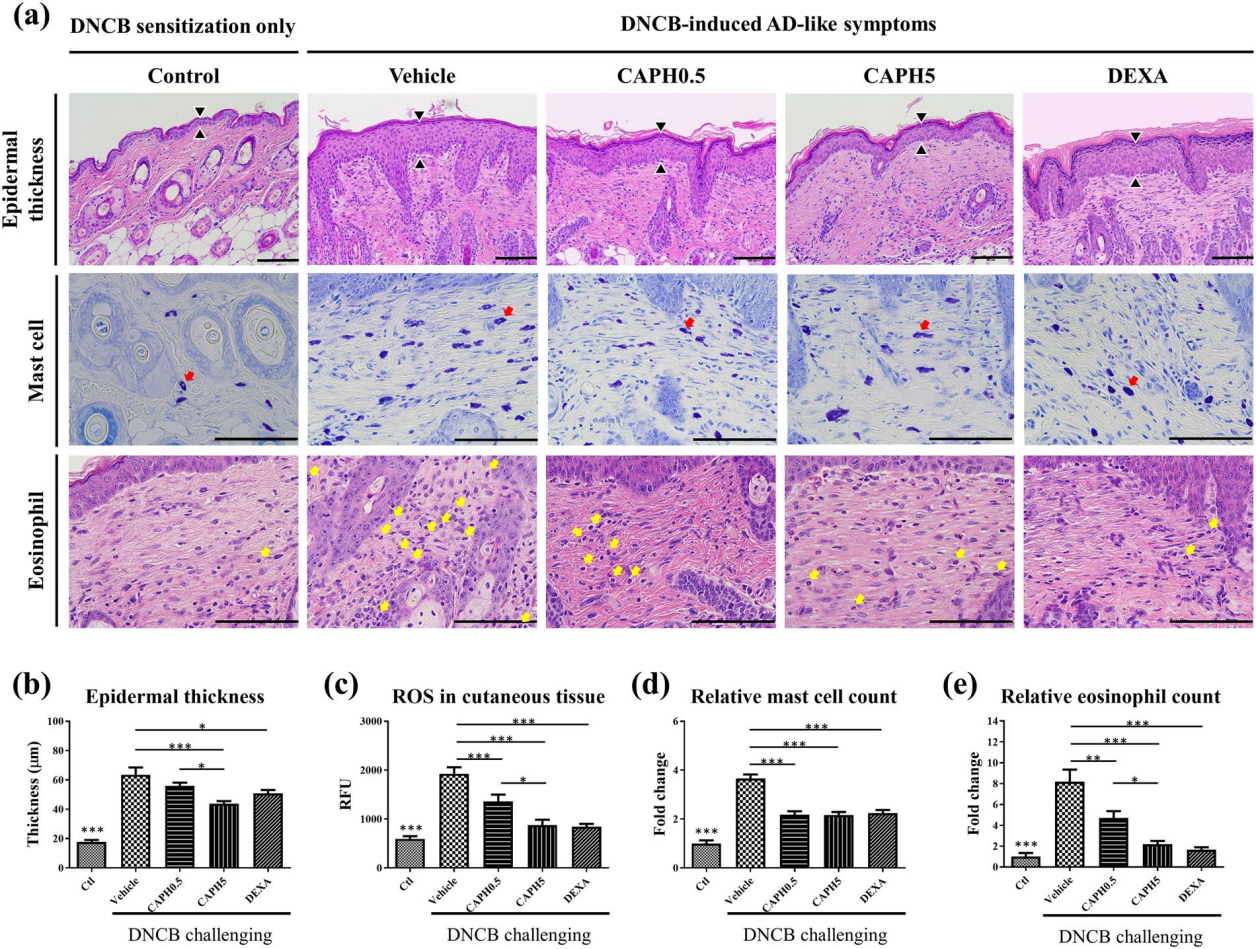
Effect of CA-PH on cutaneous histopathological observations and reactive oxygen species levels of DNCB-induced atopic dermatitis-like phenotypes in BALB/c mice. Representative histologic findings of cutaneous tissue sections stained with hematoxylin and eosin (**a**, upper and lower panels, bar: 100 µm) or toluidine blue (**a**, mid panel, bar: 100 µm). Epidermal thickness was measured in hematoxylin and eosin stained section (black arrowhead). Infiltrative mast cells (red arrow) and eosinophils (yellow arrow) were observed in the upper dermis. The thickness of epidermis (**b**), reactive oxygen species (ROS) in cutaneous tissue (**c**), the number of mast cells (**d**), and eosinophils (**e**) were quantified and compared among the groups (data are presented as the mean ± SE; *p < 0.05, **p < 0.01, and ***p < 0.001).

Histologic evaluation of the dorsal cutaneous tissue revealed that treatment of DNCB on the skin led to a cutaneous lichenification, characterized by epidermal thickening and recruitment of immune cells (mast cells and eosinophils) (Fig. 4a). CA-PH relieved DNCB effects in cutaneous tissue. First, CA-PH prevented cutaneous lichenification (Fig. 4b). The epidermal thickness significantly increased in vehicle-treated mice (17.66 ± 1.37 µm *vs*. 63.52 ± 4.95 µm; *p* < 0.001), which significantly reduced by topical application of 5 mM CA-PH (43.80 ± 1.76 µm; *p* < 0.001). The epidermis thickness also decreased in the 0.5 mM CA-PH group (55.89 ± 2.26 µm), but there was no statistical significance. ROS quantification in cutaneous tissue showed that ROS level was increased significantly in mice applied with DNCB and vehicle compared to that in the control group (*p* < 0.001) (Fig. 4c). This indicates that oxidative stress was increased in DNCB-induced AD-like phenotype mice. Nonetheless, CA-PH treatment significantly reduced the oxidative stress level in the CA-PH-treated groups dose dependently (Fig. 4c).

### CA-PH reduced mast cell and eosinophil infiltration

In terms of mast cell and eosinophil infiltration in skin, treatment of DNCB on the skin led to recruit immune cells (mast cells and eosinophils) (Fig. 4a). The inflammatory cells infiltrated in the dermis layer were induced by DNCB treatment with statistically significance (mast cell: 3.66 ± 0.16 folds; eosinophil: 8.17 ± 1.16 folds; *p* < 0.001 compared to the control group). CA-PH prevented the DNCB-induced effect (Fig. 4d,e).

On the other hand, in CA-PH-treated groups (CA-PH 0.5 mM and CA-PH 5 mM groups), mast cells and eosinophils were less infiltrative than those in the vehicle-treated group based on the toluidine blue and hematoxylin and eosin staining. In the case of mast cells, in each group, infiltrative cells were 2.18 ± 0.14 and 2.16 ± 0.13 folds to control groups of CA-PH 0.5 mM and CA-PH 5 mM, respectively, which were significantly lower than that in the vehicle group (3.66 ± 0.16 folds to control group; *p* < 0.001). CA-PH also attenuated eosinophil infiltration in dermis. CA-PH treatment (0.5 mM and 5 mM) reduced eosinophils to 4.71 ± 0.66 and 2.17 ± 0.32 folds to control, respectively, which was significantly lower than that in the vehicle group (8.17 ± 1.16 folds of control). Therefore, CA-PH prevented cutaneous lichenification and mast cell and eosinophil infiltration in dermis.

### CA-PH restored the skin barrier function-related protein level

**Figure 5:**
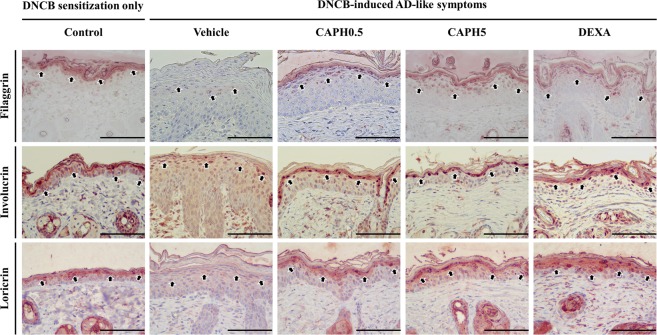
Effect of CA-PH on skin barrier function-related proteins in DNCB-induced atopic dermatitis-like phenotypes in BALB/c mice. Representative histologic section findings of mouse cutaneous tissue immunostained with filaggrin (upper panels), involucrin (mid panels), and loricrin (lower panels) at Day 28. Each protein was immunostained with dark brown color (indicated by black arrows; scale bar, 100 μm). CA-PH application group showed higher immunostaining intensity of filaggrin, involucrin, and loricrin than those of the vehicle-treated group.

The reduced skin barrier-related protein level in epidermis, manifested in the vehicle treated mouse skin tissue, was restored by CA-PH application (Fig. 5). The cutaneous immunohistochemistry specimens of the CA-PH-treated groups showed higher intensity in filaggrin, involucrin, and loricrin than those of DNCB-induced AD-like skin lesion.

### Inflammatory cytokines, proteins, and serum IgE levels in cutaneous tissue were reduced in CA-PH-treated mice

**Figure 6:**
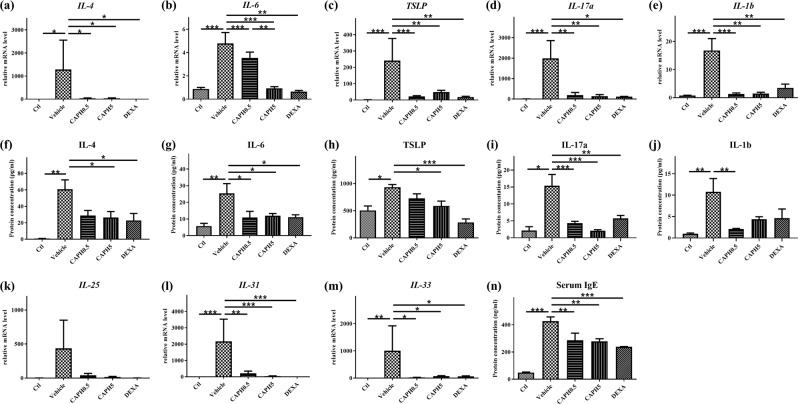
Effect of CA-PH on cutaneous cytokine mRNA, protein, and serum IgE levels of DNCB-induced atopic dermatitis-like phenotypes in BALB/c mice. The mRNA levels of inflammatory cytokines (interleukin (IL)-4, IL-6, thymic stromal lymphopoietin (TSLP), IL-17a, IL-1b, IL-31, and IL-33) were significantly elevated in the vehicle-treated group compared to those in the control group; these levels were reduced by CA-PH treatment (**a–e**,**l**,**m**). Each protein level was significantly elevated in the vehicle-treated group compared to that in the control group; the protein levels were also reduced by CA-PH treatment (**f–j**). The elevated serum IgE levels by DNCB challenging in mice decreased by CA-PH treatment prominently (**n**). (data are presented as the mean ± SE; *p < 0.05, **p < 0.01, and ***p < 0.001).

We examined cytokines and proteins in dorsal skins and serum IgE levels at Day 28. We compared the relative mRNA levels of interleukin (IL)-4, IL-6, TSLP, IL-17a, IL-1b, IL-25, IL-31, and IL-33 among the five groups. In addition, we quantified the protein levels of IL-4, IL-6, TSLP, IL-17a, and IL-1b. Noticeably, the mRNA levels of inflammatory cytokines were increased in the dorsal tissue of mice in the DNCB-induced AD-like symptom group than those in the control group (Fig. 6a–e,l,m; *p* < 0.05). Topical CA-PH treatment significantly reduced the mRNA levels of IL-4, IL-6, TSLP, IL-17a, IL-1b, IL-31, and IL-33 (*p* < 0.05). However, mRNA level of IL-25 in dorsal skin was not significantly different among the groups (Fig. 5k). Further, the protein levels of IL-4, IL-6, TSLP, IL-17a, and IL-1b also were increased in the dorsal cutaneous tissue of mice in the DNCB-induced AD-like symptom group than those in the control group (Fig. 6f–j; *p* < 0.05), and topical CA-PH treatment significantly reduced the protein levels of IL-4, IL-6, TSLP, IL-17a, and IL-1b (*p* < 0.05). On the other hand, the serum IgE level significantly increased in the DNCB-induced AD-like symptom group (*p* < 0.001), which was partly reduced by topical treatment of CA-PH (*p* < 0.01) (Fig. 6n).

## Discussion

CA-PH exhibited the highest activity in the free radical scavenging test and the lipid peroxidation inhibition test among many caffeic acid dipeptide derivatives^19,20^. That’s why we selected and applied CA-PH on the DNCB-induced  AD-like phenotypes in BALB/c mice, demonstrating that topical administration of CA-PH could improve AD-like manifestation in a DNCB-induced mouse model. The effects of CA-PH on the three major pathogenic factors of AD: 1) pruritus, 2) skin barrier abnormalities, and 3) immunologic dysregulation were identified^3^.

Pruritus is one important clinical feature and major diagnostic criterion of AD^25^. Controlling pruritus is important to treat AD in order to interrupt the ‘itch-scratch cycle’; itchiness causes scratch, which makes the lesion be more itchy and eczematous. Scratching behavior aggravates the AD severity, lowers the quality of life, and causes psychological stress^26^.

CA-PH treatment considerably reduced scratching duration (Fig. 3c). At the same time, mRNA level of IL-31, a pruritogenic cytokine^27^, was significantly reduced by CA-PH (Fig. 6l). CA-PH could attenuate pruritic symptoms by scavenging free radicals effectively. In addition, caffeic acid itself has been reported to have anti-pruritic effects by inhibiting histamine-dependent (histamine receptor 1) or histamine-independent (mas-related G-coupled protein receptor member A3) itching pathway^22^.

Skin barrier is disrupted in AD. The barrier defect makes it easy for allergens or irritants to penetrate the skin and induce immunologic response^2^. Restoring the disrupted skin barrier is important to prevent AD development^28^. The disrupted skin barrier function is reflected by increased TEWL measurements, epidermal lichenification, and decreased skin barrier-related protein levels^29^. In this study, increased TEWL, prominent epidermal lichenification, reduced filaggrin, involucrin, and loricrin expression in epidermis were observed in BALB/c mice with DNCB-induced AD-like phenotypes compared to the control.

CA-PH-treated mice manifested significantly reduced TEWL values, relatively normal epidermis in histologic examination, and restored filaggrin, involucrin, and loricrin levels in epidermis (Figs. 3–5). The stratum corneum is the most important functional part as a barrier of skin. Oxidative stress of proteins in the stratum corneum disrupts the skin barrier function and exacerbates AD^30^. In addition, ROS reduces the production of skin barrier-related proteins such as filaggrin downregulation in keratinocytes^31^. The antioxidant effect of CA-PH may affect the stratum corneum and keratinocytes, resulting in skin barrier function recovery. In addition, caffeic acid itself promotes involucrin expression^32^.

Immunologic dysregulation is a major factor in pathogenesis of AD. TSLP can initiate cutaneous allergic response in AD. TSLP overexpression in transgenic mouse skin showed AD-like manifestation with dermal inflammatory cell (Th2) infiltration and elevated serum IgE levels^33^. TSLP is highly expressed in keratinocytes of human AD patients, activating dendritic cells and causing Th2 responses^34^. IL-1β acts a mediator of AD phenotype by inducing TSLP^35^. Along with TSLP, IL-33 and IL-25 are other tissue-derived cytokines that are crucial in AD by promoting Th2 cell response^36,37^. Effector cytokines for Th2 response in AD include IL-4, IL-6 and IL-31, which augment Th2 response^38–40^. In addition, IL-17a, one of the Th17 cytokines, could also mediate Th2 immune responses^41^. IL-31 and IL-33 activate the eosinophil-fibroblast interaction in AD, inducing tissue damage^42^. Increased dermal mast cell and eosinophil infiltration is well characterized in AD skin tissue, and their activation contribute to AD^43–45^.

CA-PH treatment normalized the Th2-deviated immunologic dysregulation in DNCB-induced AD-like phenotypes in BALB/c mice. CA-PH ameliorated the effect of DNCB by significantly reducing the mRNA and protein levels of IL-4, IL-6, TSLP, IL-17a, and IL-1b as well as the mRNA expression levels of IL-31 and IL-33 in the mouse dorsal skin. From histologic cellular examination, we found that significantly increased mast cell and eosinophil infiltration was notably reduced by topical application of CA-PH (Fig. 4).

We demonstrated that CA-PH efficiently exerted relieving effects on DNCB-induced AD-like phenotypes in BALB/c mice, relieving pruritus, restoring skin barrier, and normalizing immunologic dysregulation. Thus, CA-PH may be a promising and safe candidate for treating AD in the future.

## Materials and Methods

### Ethical approval

The animal experimental protocol was approved by Seoul National University Hospital Institutional Animal Care and Use Committee (No.17-0174-S1A0). All experiments were performed in accordance with the approved experimental protocol.

### Animals, induction of AD, and treatment

To induce AD-like cutaneous condition in mouse, we used cutaneous DNCB sensitization and challenging in BALB/c mice. The experimental mouse model can elicit AD-like immunologic and pathophysiological features^46,47^. Twenty 5-week-old female BALB/c mice were purchased from Orient Bio Inc. (Seongnam, Republic of Korea) and housed under semi specific pathogen-free conditions with individual ventilated cages (24 ± 2 °C with a 12-h light-dark cycle). They were fed on standard laboratory chow and water *ad libitum*.

The detailed schedule of the experiment is shown in Fig. 2. After 1 week of acclimation period, the dorsal area of mice was shaved and depilated (5 cm^2^). At 1 day (designated as Day -7) and 4 days (designated as Day -4) after shaving and depilation, 200 µL 1% DNCB dissolved in an acetone:olive oil mixture (3:1 vol/vol) was applied on the dorsal skin of the mice (i.e., cutaneous DNCB sensitization).

The cutaneous DNCB sensitized mice were divided into five groups: (1) DNCB sensitization only, (2) DNCB-induced AD + vehicle, (3) DNCB-induced AD + CA-PH 0.5 mM, (4) DNCB-induced AD + CA-PH 5 mM, and (5) DNCB-induced AD + topical dexamethasone 25 µM. In group (1) as a control group, mice were observed until the end of experiment (designated as Day 27) without any treatment. In group (2)–(5), 200 µL 0.4% DNCB in an acetone:olive oil mixture (3:1 vol/vol) was applied to the dorsum three times a week for 4 weeks (day 0–27) (cutaneous DNCB challenging). Vehicle solution was topically applied to the mouse dorsal skin of group (2) daily for 2 weeks (Day 14–27). CA-PH dissolved in vehicle (0.5 mM or 5 mM) was topically applied to the dorsum of mouse (200 µL/mouse/day) in group (3) and (4) daily for 2 weeks (Day 14–27). CA-PH was prepared according to the reported procedure^18,20^. The mice in group (5) were treated daily by dexamethasone (25 µM in phosphate-buffered saline, 200 µL/mouse/day; Day 14–27), as a positive control group.

### Quantification of DNCB-induced dermatitis score and scratching behavior

We investigated the relieving effect of CA-PH on DNCB-induced AD-like morphology in BALB/c mice by two indicators: dermatitis severity score and scratching duration. The dermatitis severity score was measured and compared among the five groups at Day 28 according to the criteria previously described, with a slight modification^12^. Briefly, the score was defined as the sum of the discrete scores graded as 0 (none), 1 (mild), 2 (moderate), and 3 (severe) for each of four signs: erythema, edema/papulation, excoriation, and scaling/dryness. A total dermatitis score ranged from 0 to 12. The elements of dermatitis score evaluation are widely used to evaluate the severity of AD^48^. To quantify pruritus symptom, we measured the duration of scratching of the body with their hind paws by the recorded video of the mice for 10 min at Day 28. The dermatitis score and the scratching duration were measured by two independent observers (J.O. and J.W.K.). The results were determined as the average of the two measurements.

### Trans-epidermal water loss (TEWL) measurement

TEWL is the value of water loss across the stratum corneum measured non-invasively *in vivo*. It was measured at Day 28. TEWL in mouse dorsal skin was measured under specific conditions at 24 °C and 50–55% humidity by using a skin water evaporation recorder, Gpskin Barrier (Gpskin, Seoul, Republic of Korea)^49^. The probe was placed at the center of the shaved dorsum area of mouse to record the TEWL value in g/m^2^/h. The statistical value was expressed in terms of fold change compared to the control group.

### Histopathological examination

At Day 28, mice were anesthetized and sacrificed to obtain sample of dorsal skin and serum. Excised dorsal cutaneous tissue were fixed in 4% formalin for 18 h and embedded in paraffin. After that, 4-µm thickness sections (of skin tissue) were prepared and stained with hematoxylin and eosin (H&E) to evaluate the thicknesses of epidermis and eosinophil count. For evaluating mast cell infiltration in the cutaneous tissue, 0.01% toluidine blue staining was used. The mean numbers of mast cells were obtained by averaging the number observed at five microscopic fields of view (sized 200 µm × 250 µm) per each mouse. Immunohistochemical staining (method) for the filaggrin, involucrin, and loricrin were used to evaluate the CA-PH effects on the skin barrier function-related proteins in the epidermis^50–52^.

### ROS quantification

ROS in the cutaneous tissue was quantified using the *in vitro* ROS/reactive nitrogen species assay kit OxiSelect (Cell Biolabs, San Diego, CA) following the instructions provided by the manufacturer.

### Levels of tissue cytokines, proteins, and serum IgE

At Day 28, we measured the length and weight of the spleen for evaluating splenomegaly, which indicated immune abnormality^53^. The relative mRNA levels of tissue TSLP and Th2 cytokines among the mice were measured by real time-polymerase chain reaction (RT-PCR) analysis (Applied Biosystems, Foster City, CA, USA). Total RNA was isolated from the mouse dorsal tissues by using RNAiso Plus reagent (Takara Bio, Shiga, Japan). The RNA was reverse-transcribed by using First Strand cDNA Synthesis kit (Fermentas, Sankt Leon-Rot, Germany) according to the manufacturer’s instruction and used for PCR using the primers listed in Supplementary Information. The amplification protocol was as follows: 3 min at 94 °C, 30 s at 94 °C, 30 s at 60 °C, 45 s at 72 °C, and 1 min at 72 °C for 35 cycles. The mRNA level of each target gene was normalized to mouse 36B4. We excised mouse dorsal cutaneous tissues (5 mm × 5 mm - sized) for preparing tissue lysates using cell lysis buffer in the kit (Bio-rad #171304011, USA) and TissueLyser II (QIAGEN, Germany), as manufacturer’s instructions. Individual cytokine protein levels in tissue lysates were quantified by Bio-Plex Pro Mouse Cytokine & Chemokine Assays on Bio-Plex® multiplex system (Bio-rad, USA). In case of TSLP, we used mouse TSLP enzyme-linked immunosorbent assay kit (Abcam #ab155461, UK), as manufacturer’s instructions. Serum IgE levels were measured using enzyme-linked immunosorbent assay kits (Abcam, #ab157718, UK), as per manufacturers’ direction.

### Statistical analysis

All statistical analyses were performed using the SPSS 22.0 software (IBM, Armonk, NY, USA). The results were expressed as the mean ± standard error of means. The results of multiple group analysis were analyzed using One-Way analysis of variance (ANOVA) followed by Tukey’s significant difference test. Data were representative of at least two independent experiments. P-values <0.05 were considered statistically significant.

## Supplementary information

Supplementary information.
