# Supplementary material for: Caffeoyl–Pro–His amide relieve DNCB-Induced Atopic Dermatitis-Like phenotypes in BALB/c mice
Source: Sci Rep. 2020 May 21;10:8417. doi: 10.1038/s41598-020-65502-2 (PMC7242424; doi:10.1038/s41598-020-65502-2)
Supplement: Supplementary file 1 — Supplementary information. [file 41598_2020_65502_MOESM1_ESM.pdf]

Manuscript title:

Caffeoyl–Pro–His amide relieve DNCB-Induced Atopic Dermatitis-Like phenotypes in BALB/c mice

Running title: Caffeoyl–Pro–His amide improves Atopic Dermatitis

Full Names of Authors and Affiliations:

Sunhyae Jang, PhD[1¶] Jungyoon Ohn, MD[1¶] Ji Won Kim, MD[1] So Min Kang, PhD[2] Dasom Jeon, MS[2] Chan Yeong Heo, MD,PhD[2] Yoon-Sik Lee, PhD[3] Ohsang Kwon, MD, PhD [1] Kyu Han Kim, MD, PhD[1*]

[1] Laboratory of Cutaneous Aging and Hair Research, Clinical Research Institute, Seoul National University Hospital, Seoul, Republic of Korea; Institute of Human Environment Interface Biology, Seoul National University Seoul, Republic of Korea; Department of Dermatology, College of Medicine, Seoul National University, Seoul, Republic of Korea

[2] Department of Plastic and Reconstructive Surgery, College of Medicine, Seoul National University, Seoul, Republic of Korea; Institute of Human Environment Interface Biology, Seoul National University, Seoul, Republic of Korea; Department of Plastic and Reconstructive Surgery, Seoul National University Bundang Hospital, Gyeonggi-do, Republic of Korea

[3] School of Chemical and Biological Engineering, Seoul National University, Seoul, Republic of Korea

¶ Both authors contributed equally to this work

Correspondence and Reprint Request:

Kyu Han Kim, M.D., Ph.D.

Department of Dermatology, Seoul National University College of Medicine, 101 Daehak-ro, Jongno-gu, Seoul 03080, South Korea

E-mail: kyuhkim@snu.ac.kr

Supplementary Information. PCR primer sequences for mRNA quantification

Item	Forward or reverse	Sequence
mouse IL-4	F	5’-acaggagaagggacgccat-3’
	R	5’-gaagccgtacagacgagctca-3’
mouse IL-25	F	5’-cggaggagtggctgaagtggag-3’
	R	5’-atgggtaccttcctcgccatg-3’
mouse IL-31	F	5’-tcggtcatcatagcacatctggag-3’
	R	5’-gcacagtccctttggagttaagtc-3’
mouse IL-33	F	5’-gatgggaagaagctgatggtg-3’
	R	5’-ttgtgaaggacgaagaaggc-3’
mouse TSLP	F	5’-cggatggggctaacttaca-3’
	R	5’-tcctcgatttgctcgaactt-3’
mouse IL-6	F	5’-gaggataccactcccaacagacc -3’
	R	5’-aagtgcatcatcgttgttcataca - 3’
mouse IL-1b	F	5’-tgtaatgaaagacggcacacc-3’
	R	5’-tcttctttgggtattgcttgg-3’
mouse IL-17a	F	5’-atccctcaaagctcagcgtgtc-3’
	R	5’-gggtcttcattgcggtggagag-3’
